# Scopolamine regulates the osteogenic differentiation of human periodontal ligament stem cells through lactylation modification of RUNX2 protein

**DOI:** 10.1002/prp2.1169

**Published:** 2024-01-23

**Authors:** Ying Wu, Pan Gong

**Affiliations:** ^1^ Department of Periodontics Affiliated Stomatology Hospital of Guangzhou Medical University Guangzhou China; ^2^ Guangdong Engineering Research Center of Oral Restoration and Reconstruction Guangzhou China; ^3^ Guangzhou Key Laboratory of Basic and Applied Research of Oral Regenerative Medicine Guangzhou China; ^4^ Department of Stomatology Affiliated Cancer Hospital & Institute of Guangzhou Medical University Guangzhou China

**Keywords:** lactylation, osteogenic differentiation, PDLSC, RUNX2, scopolamine

## Abstract

Periodontal ligament stem cells (PDLSCs) are important mesenchymal stem cells contributing to regenerating lost periodontal tissues and repairing bone defects. Studies on the molecular mechanism affecting the osteogenic differentiation of PDLSCs are necessary. Scopolamine (SCO) is known as a regulator of neural cell damage. The focus of the current study is on unveiling the role of SCO‐mediated molecular mechanism in the osteogenic differentiation of PDLSCs. Through CCK‐8 assay and LDH detection, we confirmed that SCO enhanced the viability of PDLSCs. Moreover, we determined that SCO induced the PDLSCs osteogenic differentiation, according to data of ALP activity measurement and ARS staining. Mechanistically, we performed western blot and identified that SCO could promote the lactylation of runt‐related transcription factor 2 (RUNX2). We also found through rescue assays that knockdown of RUNX2 could reverse the effect of SCO treatment on the osteogenic differentiation of PDLSCs. Further mechanism investigation revealed that lactylation of RUNX2 at K176 site enhances the protein stability of RUNX2 through deubiquitination. Collectively, our present study unveils that SCO stabilizes RUNX2 to promote the osteogenic differentiation of PDLSCs through the lactylation modification of RUNX2.

## Graphical abstract

Scopolamine regulates the osteogenic differentiation of human periodontal ligament stem cells through modulating the lactylation and ubiquitination modification of RUNX2 protein

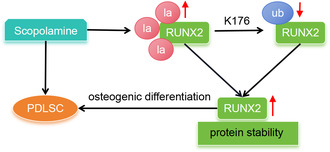

**Abbreviations**
MSCs: mesenchymal stem cells
PDLSCs: periodontal ligament stem cells
RUNX2: runt‐related transcription factor 2
SCO: scopolamine

## 1 INTRODUCTION

Periodontal ligament stem cells (PDLSCs) derive from the periodontal ligament, which belongs to the group of mesenchymal stem cells (MSCs).^1^, ^2^ PDLSCs are capable of regenerating lost periodontal tissues.^3^, ^4^, ^5^ The comparison between the osteogenic potential of PDLSCs in basal and differentiating culture media is of great benefit in exploring the mechanism of human periodontal disease.^6^ Previous studies have demonstrated that exogenous additives can induce osteogenic differentiation of PDLSCs, such as short peptides,^7^ which can be used for future regenerative cell therapy. In addition, the repairing ability of PDLSCs has been reported in the orthodontic tooth movement progress^8^, ^9^ and periodontitis‐induced bone destruction^10^ and bone defect.^11^, ^12^ Studies on the molecular mechanism underlying the osteogenic differentiation of PDLSCs are needed.^13^

Scopolamine (SCO) is widely used as an inducer of nerve system damage for in vivo animal models, causing memory loss and cognitive impairment.^14^, ^15^, ^16^ Functionally, SCO can regulate PC12 cell damage and energy metabolism^16^; moreover, it can inhibit alveolar bone loss in rats.^17^ However, the specific role of SCO in modulating the osteogenic differentiation of PDLSCs is rarely reported. The focus of the current study is on the regulating functions of SCO in the osteogenic differentiation of PDLSCs as well as the corresponding regulatory mechanism.

Lactylation modification is known as a post‐translational regulatory mode, altering the protein stability.^18^ Runt‐related transcription factor 2 (RUNX2) is an osteogenic gene, involving in the osteogenic differentiation of PDLSCs.^19^, ^20^, ^21^ To date, it remains unclear whether SCO affects the lactylation‐mediated stability of RUNX2 in PDLSCs.

To summarize, the current study makes an investigation on the role of SCO‐mediated lactylation of RUNX2 in the osteogenic differentiation of PDLSCs.

## 2 MATERIALS AND METHODS

### 2.1 Cell culture and treatment

Human PDLSCs were procured from BeNa culture collection (Beijing, China). For cell culture, PDLSCs were placed in Dulbecco's modified Eagle medium (DMEM) (Sigma, USA) containing 10% FBS (Hyclone, Logan, UT, USA). For incubation, the culture dishes were maintained in an incubator at 37°C and 5% CO_2_.

PDLSCs were treated with low (25 nM), middle (50 nM), or high (100 nM) concentrations of scopolamine (SCO, Sigma‐Aldrich, St. Louis, MO, USA) to screen for suitable concentration.

For protein stability detection, PDLSCs were treated with 50 μg/mL of CHX (MedChemExpress, NJ, USA) for 0, 6, 12, and 24 h.

### 2.2 Cell transfection

Short hairpin RNAs (shRNAs) targeting RUNX2 (sh‐RUNX2#1, sh‐RUNX2#2) and corresponding negative control shRNA (sh‐NC) were synthesized by RiboBio (Guangzhou, China). PDLSCs with confluence reached to 50%–60% were transfected with shRNAs for 48 h by using the Lipofectamine 2000 reagent (Invitrogen, CA, USA).

### 2.3 Reverse transcription and real‐time quantitative polymerase chain reaction (RT‐qPCR)

Total RNA extracted from indicated PDLSCs using Trizol Reagent (Invitrogen) was subjected to reverse transcription by using PrimeScript RT Reagent Kit (Takara, Tokyo, Japan) for complementary DNA (cDNA) generation. qRT‐PCR was conducted by using the SYBR Prime Script RT‐PCR Kit (Takara), as instructed by the manufacturer's protocol. The relative mRNA expression was calculated with the 2^−ΔΔCt^ method by taking GAPDH as the internal control.

### 2.4 Immunoprecipitation (IP)

PDLSCs treated with or without SCO were lysed with RIPA buffer. Next, the lysates were pretreated with 50 μL of protein A/G immune magnetic beads (Bimake, Houston, TX, USA) and were immunoprecipitated with antibodies obtained from Abcam (Cambridge, MA, USA), including anti‐RUNX1, anti‐RUNX2, anti‐Osx, anti‐ONT, anti‐OPN, anti‐OCN, anti‐OPG, anti‐BMP2, anti‐BMP7, and anti‐COL1A1. Finally, the lactylation level of RUNX2 was detected with western blot using an l‐lactyllysine (PTM Biolabs, Chicago, IL) antibody.

### 2.5 Western blot

Total protein was isolated from indicated PDLSCs using RIPA buffer (Beyotime, China). Protein samples were loaded on a 10% sodium dodecyl sulfate–polyacrylamide gel electrophoresis (SDS–PAGE) and then transferred to a polyvinylidene fluoride (PVDF) membrane. After washing, the membrane was incubated overnight with primary antibodies against OCN (1/1000, Abcam), OPG (1/1000, Abcam), RUNX2 (1/1000, Abcam), and the internal control anti‐GAPDH (1/1000, Abcam) at 4°C. Next, the membrane was further incubated at room temperature with the second antibody (1/2000, Abcam) for 2 h. Finally, blots were visualized and photographed with an optical luminescence instrument (GE, USA).

### 2.6 Ubiquitination assay

PDLSCs with or without RUNX2 mutation of K176 site (WT or K176R) were lysed with RIPA buffer. Next, the lysates were pretreated with 50 μL of protein A/G immune magnetic beads (Bimake, Houston, TX, USA) and were immunoprecipitated with anti‐RUNX2 antibody. The ubiquitinated RUNX2 was detected with western blot using an anti‐ubiquitin antibody (1:1000, Abcam).

### 2.7 Cell counting kit‐8 (CCK‐8) assay

The effect of SCO on the viability of PDLSCs was evaluated by the CCK8 (Dojindo Laboratories, Kumamoto, Japan) assay, as instructed by the manufacturer's protocol. In brief, PDLSCs were incubated in 96‐well plates at a density of 3 × 10^3^ cells/well along with low, middle, or high concentrations of SCO. Forty‐eight hours later, each well was added with 10 μL CCK8 solution for 2 h incubation. Finally, a microplate reader was used to measure the absorbance at a wavelength of 450 nm.

### 2.8 Detection of lactate dehydrogenase (LDH) release

To detect the effect of SCO on the necrotic cell death condition of PDLSCs, the LDH level was measured. Briefly, PDLSCs were seeded into 96‐well plates at a density of 5 × 10^4^ cells/well and then incubated for 24 h. After that, the supernatant was collected and incubated with an LDH cytotoxicity assay kit. Results were obtained by measuring the absorbance at 560 nm with a microplate reader. Finally, the percentage of LDH release of treated PDLSCs was calculated and compared with the control group.

### 2.9 Detection of alkaline phosphatase (ALP) activity

PDLSCs (2 × l0^5^ cells) were seeded in each well of 6‐well plates and incubated in the osteogenic inductive medium that consisted of DMEM, 10% FBS, 10 mM β‐glycerophosphate, 10 mM dexamethasone, and 50 mg/L ascorbic acid. Meanwhile, each well was added with the middle concentration of SCO or specific shRNAs. Two weeks later, cells were treated with 1% TritonX‐100 (Solarbio). And then, cells were subjected to centrifugation at 12000*g* for 10 min. The ALP activity assay kit (Jiancheng Bioengineering Institute, Nanjing, China) was applied to measure ALP activity, according to the manufacturer's instructions. Finally, a microplate reader was used to monitor the absorbance at 520 nm wavelength.

### 2.10 Alizarin Red S (ARS) staining

An ARS staining kit (Solarbio, Beijing, China) was applied to monitor the mineral deposition of indicated PDLSCs, as instructed by the manufacturer's protocol. Briefly, PDLSCs were fixed in 4% paraformaldehyde for 10 min and washed thrice with PBS. Next, PDLSCs were stained for 30 min by 40 mM ARS (Sigma‐Aldrich). The reaction was terminated by adding the distilled water. Finally, the results were observed and photographed.

### 2.11 Statistical analysis

All data were presented in the form of mean ± standard deviation (SD) by processing with GraphPad Prism 8 (GraphPad Software Inc., CA, USA). The comparison for two groups or more than two groups was performed by student's *t*‐test or one‐way ANOVA using SPSS v22.0 statistical analysis tool (IBM, CA, USA). *p* < .05 is a symbol for a statistically significant difference.

## 3 RESULTS

### 3.1 SCO enhances the viability of PDLSCs

**FIGURE 1:**
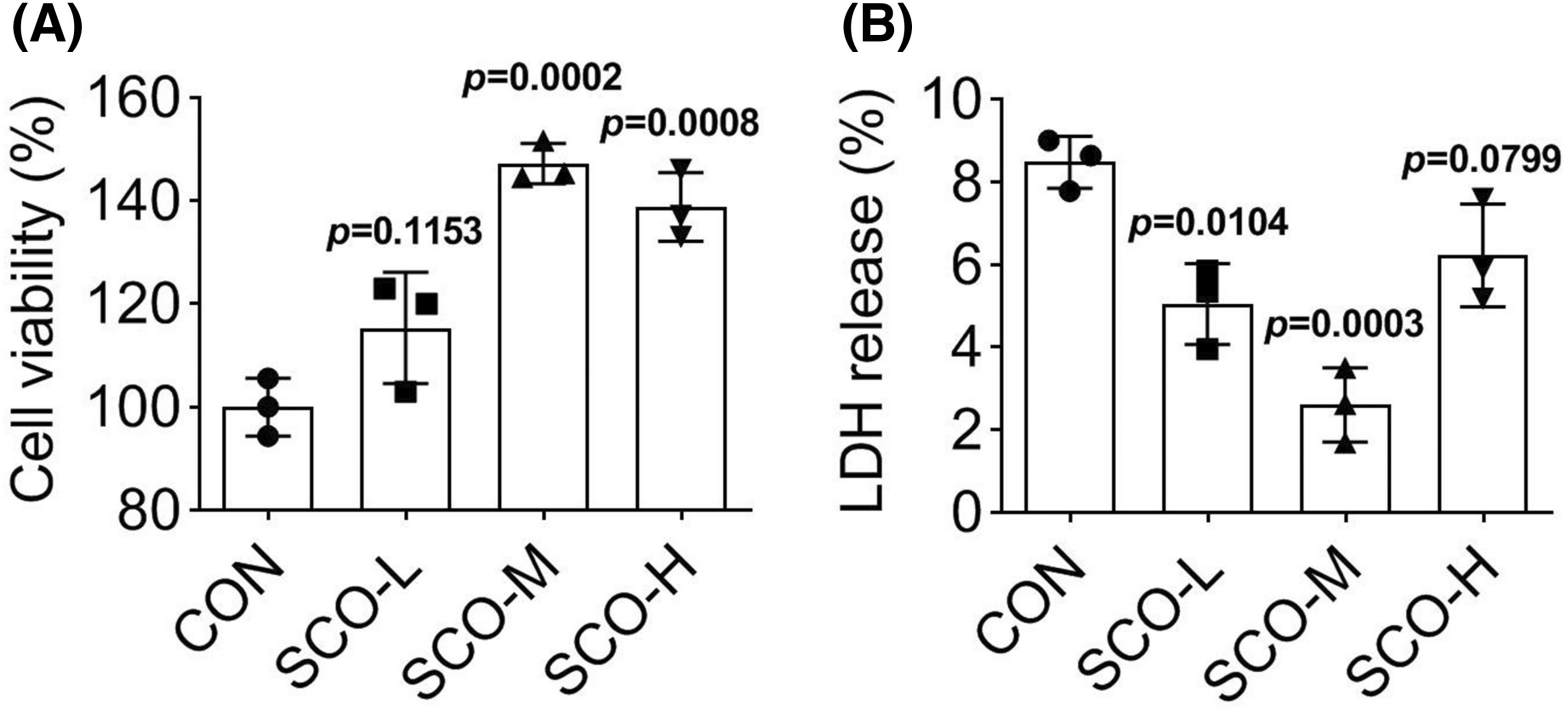
SCO enhances the viability of PDLSCs. (A) CCK‐8 assay was applied to measure the viability of PDLSCs treated with low, middle, or high concentrations of SCO (SCO‐L, SCO‐M, or SCO‐H). *p* = .002, *p* = .008 indicated data were statistically significant. *p* = .1213 indicated data were not statistically significant. (B) LDH release of PDLSCs was measured in PDLSCs treated with three different concentrations of SCO through LDH detection. *p* = .0104, *p* = .0003 indicated data were statistically significant. *p* = .0799 indicated data were not statistically significant.

In order to detect whether SCO affected the functions of PDLSCs, we used three different concentrations of SCO (low, middle, and high) to treat PDLSCs and measured cell viability through the CCK‐8 assay. According to the CCK8 data shown in Figure 1A, after 48 h culture, the viability of PDLSCs was significantly enhanced by treatment with middle or high concentration of SCO (SCO‐M or SCO‐H). Additionally, LDH detection indicated that the LDH release of PDLSCs was suppressed efficiently by treatment with low or middle concentration of SCO (SCO‐L or SCO‐M) (Figure 1B). All these results indicated that middle concentration of SCO had a greater effect on cell viability and have the best inhibitory effect on cytotoxicity. Therefore, SCO‐M was selected for the next experiments.

### 3.2 SCO promotes the osteogenic differentiation of PDLSCs

**FIGURE 2:**
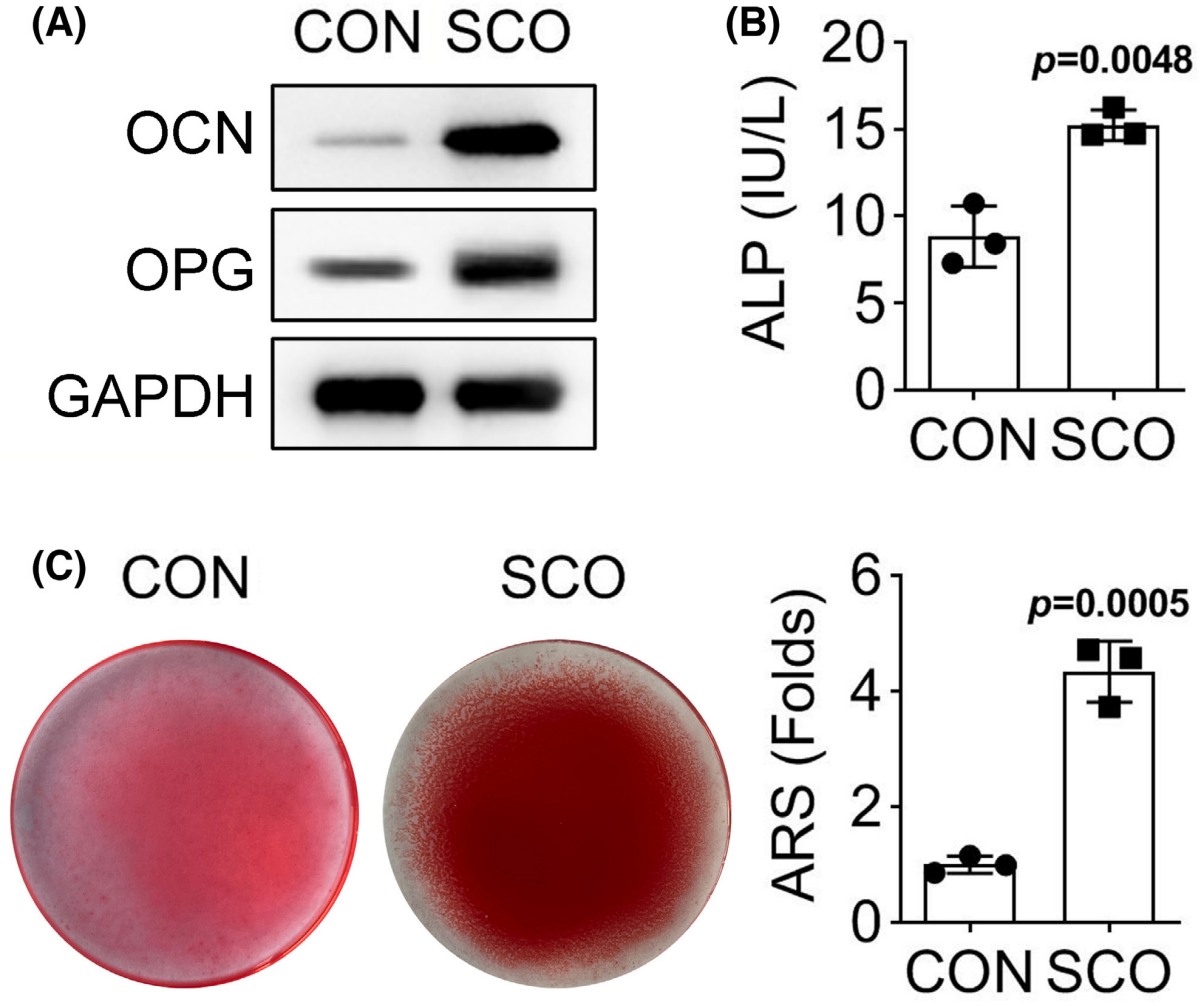
SCO promotes the osteogenic differentiation of PDLSCs. (A) The protein levels of two osteogenic genes (OCN and OPG) were measured by western blot in PDLSCs treated with SCO or control (CON). (B) ALP activity of PDLSCs was measured in PDLSCs treated with or without SCO. *p* = .0048 indicated data were statistically significant. (C) The mineral deposition of PDLSCs treated with or without SCO was detected by ARS staining. *p* = .0005 indicated data were statistically significant.

Subsequently, we explored whether SCO could regulate the osteogenic differentiation of PDLSCs. Since the middle concentration of SCO could most efficiently alter cell viability, we chose it for all subsequent experiments. We first measured the protein levels of two osteogenic genes (OCN and OPG) in PDLSCs treated with SCO or without SCO (the control group; CON). It was found that both levels of OCN and OPG were increased a lot by SCO treatment (Figure 2A). Next, the ALP activity of PDLSCs was strengthened by SCO treatment (Figure 2B), indicating that SCO promoted in PDLSCs. Furthermore, the result of ARS staining showed that the mineral deposition was accumulated in PDLSCs after being treated with SCO (Figure 2C). Therefore, we conclude that SCO treatment induces the osteogenic differentiation of PDLSCs.

### 3.3 SCO induces the lactylation of RUNX2 protein in PDLSCs

**FIGURE 3:**
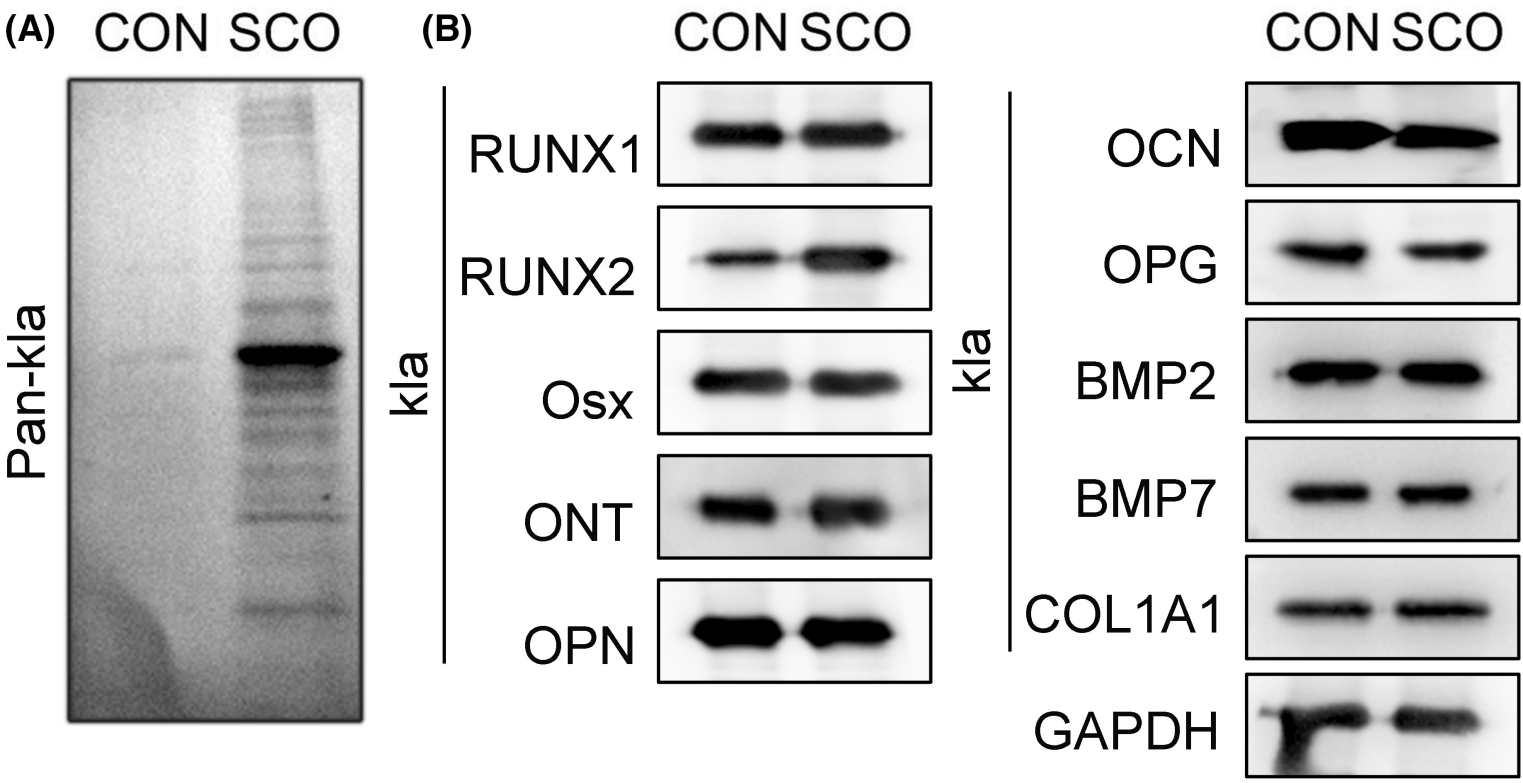
SCO induces the lactylation of RUNX2 protein in PDLSCs. (A) Total lactylation levels were measured in PDLSCs treated with or without SCO through IP‐western blot. (B) The lactylation levels of osteogenic proteins were evaluated in PDLSCs treated with or without SCO through IP‐western blot.

Lactylation is a post‐translational modification, exerting functions in various biological processes.^22^, ^23^ Here, we investigated whether the SCO altered protein lactylation in PDLSCs. Through western blot analysis of IP results, we determined that total lactylation levels were elevated in SCO‐treated PDLSCs compared with the control group (Figure 3A). Since osteogenic genes and proteins are essential to osteogenic differentiation,^24^ we measured the lactylation levels of osteogenic proteins in SCO‐treated PDLSCs. It was uncovered that the lactylation level of RUNX2 was significantly increased in PDLSCs after SCO treatment (Figure 3B). The results suggest that SCO can promote the lactylation of RUNX2 in PDLSCs.

### 3.4 Knockdown of RUNX2 reverses the effect of SCO treatment on the osteogenic differentiation of PDLSCs

**FIGURE 4:**
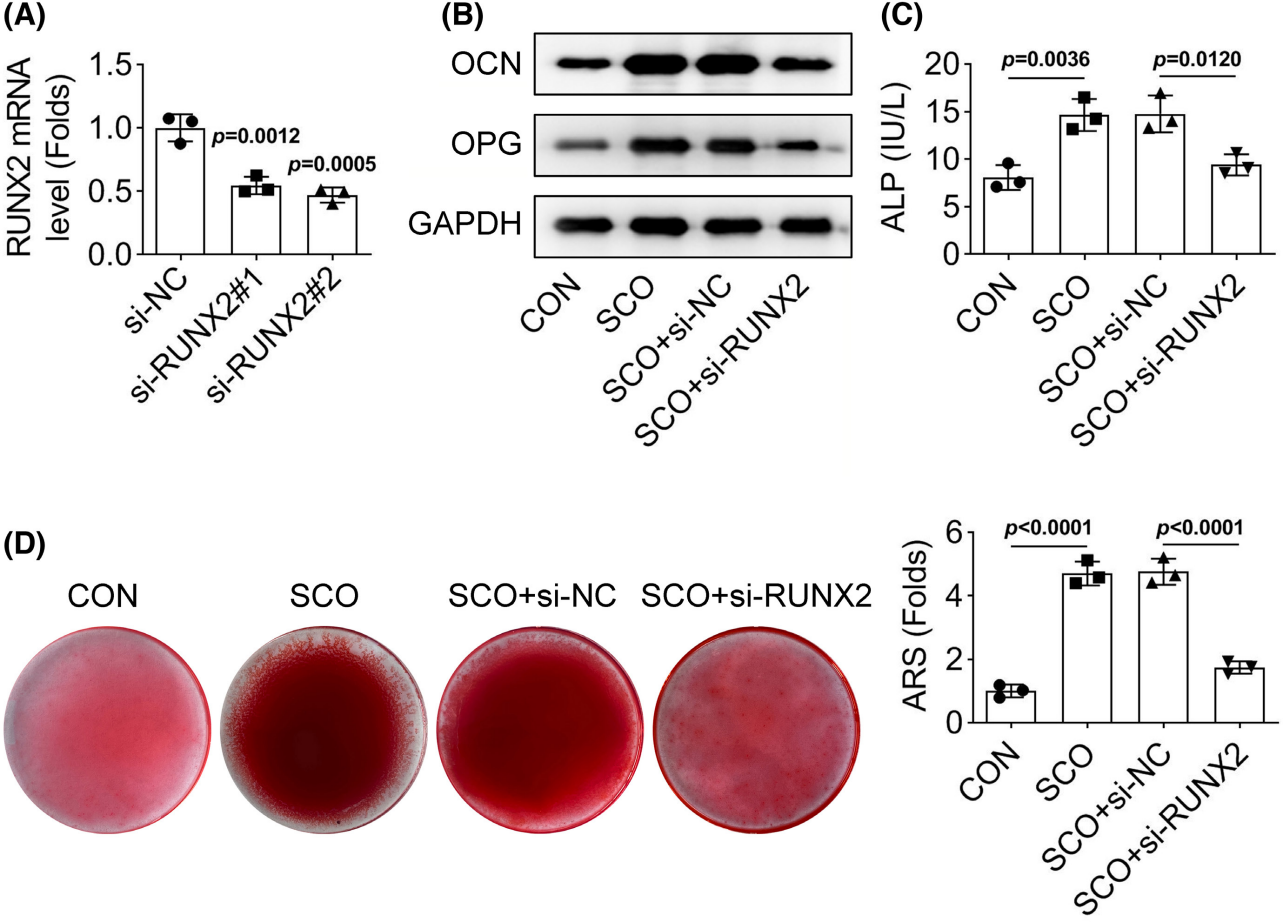
Knockdown of RUNX2 reverses the effect of SCO treatment on the osteogenic differentiation of PDLSCs. (A) RUXN2 expression was knocked down through exogenously transfecting shRNAs targeting RUNX2. The transfection efficiency was identified by RT‐qPCR analysis. *p* = .0015, *p* = .0005 indicated data were statistically significant. (B) The protein levels of OCN and OPG were detected by western blot in SCO‐treated PDLSCs after knockdown of RUNX2. (C) The ALP activity of SCO‐treated PDLSCs was evaluated after knockdown of RUNX2. *p* = .0036, *p* = .0120 indicated data were statistically significant. (D) The mineral deposition of SCO‐treated PDLSCs was evaluated by ARS staining after knockdown of RUNX2. *p* < .0001 indicated data were statistically significant.

To validate the involvement of SCO‐induced RUNX2 in the osteogenic differentiation of PDLSCs, rescue assays were carried out. Before that, RUXN2 expression was knocked down through exogenously transfecting shRNAs targeting RUNX2 (Figure 4A). Next, we detected the protein levels of OCN and OPG and identified that the increased levels of both OCN and OPG caused by SCO treatment were decreased again by RUNX2 knockdown (Figure 4B). Additionally, the ALP activity of PDLSCs strengthened by SCO treatment was weakened after knockdown of RUNX2 (Figure 4C). Meanwhile, the mineral deposition accumulated in SCO‐treated PDLSCs was reduced after knockdown of RUNX2 (Figure 4D). Hence, we confirm that SCO promotes the osteogenic differentiation of PDLSCs through RUNX2.

### 3.5 Lactylation of RUNX2 at K176 site enhances the protein stability of RUNX2 through deubiquitination

**FIGURE 5:**
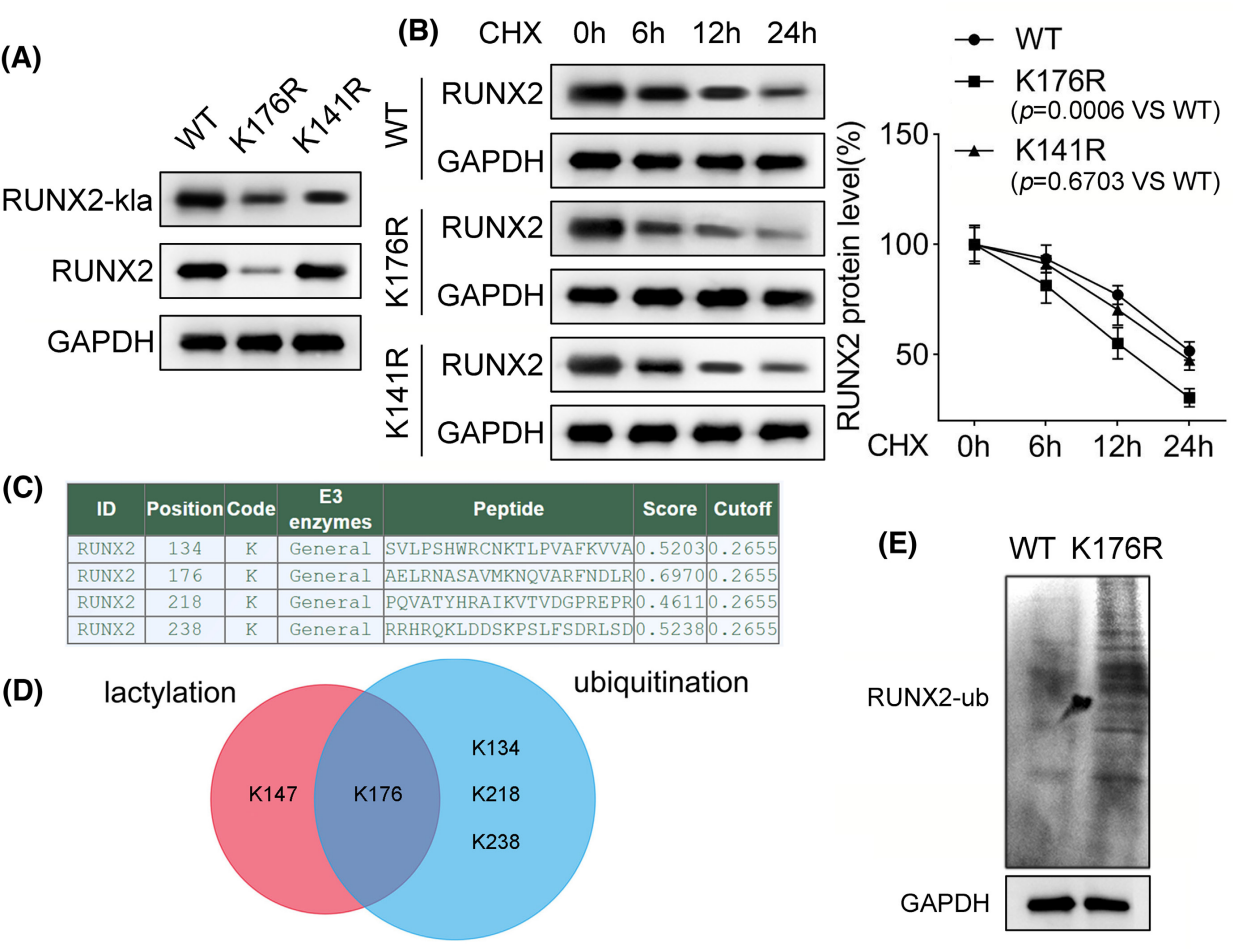
Lactylation of RUNX2 at K176 site enhances the protein stability of RUNX2 through deubiquitination. (A) The lactylation level and total protein level of RUNX2 were measured in PDLSCs after the RUNX2 mutation of two potential lactylation sites (K176R and K141R). Results were obtained using western blot analysis. (B) The half‐life profile of RUNX2 was performed in RUNX2 K176 or K141 site‐mutated PDLSCs after being treated with CHX for different time intervals. *p* = .0006 indicated data were statistically significant. *p* = .6703 indicated data were not statistically significant. (C) The potential ubiquitination sites of RUNX2. (D) K176 site of RUNX2 protein might occur both ubiquitination and lactylation through intersection. (E) The ubiquitination assay was performed in PDLSCs with or without mutation of K176 site to measure the ubiquitination level of RUNX2.

To identify the functional site, we mutated two potential lactylation sites (K176R and K141R) for further western blot analysis. The results showed that mutation of K176 site decreased the lactylation level and total protein level of RUNX2 while mutation of K141 site had no significant effects on both levels (Figure 5A). Studies have shown that the lactylation modification can enhance protein stability.^25^ Hence, we analyzed whether the lactylation of RUNX2 altered its stability. We treated PDLSCs with or without RUNX2 mutation of K176 or K141 site with CHX for half‐life profile of RUNX2. The results indicated that mutation of K176 site shortened the half‐life time of RUNX2 (Figure 5B), suggesting that the lactylation of RUNX2 at K176 site affected the stability of RUNX2. Subsequently, we analyzed whether the lactylation of RUNX2 at K176 site could change the ubiquitination of RUNX2. We next analyzed the potential ubiquitination sites of RUNX2 (Figure 5C) and found that K176 site might occur both the ubiquitination and lactylation through intersection (Figure 5D). To validate the hypothesis, we performed the ubiquitination assay in PDLSCs with or without mutation of K176 site. The result unveiled that mutation of K176 site increased the ubiquitination level of RUNX2 (Figure 5E). Therefore, we summarize that the lactylation of RUNX2 at K176 site decreases the ubiquitination level of RUNX2 to stabilize RUNX2 protein.

## 4 DISCUSSION

Our current study firstly determined the promoting effect of SCO on the viability and osteogenic differentiation of PDLSCs. Furthermore, SCO treatment could elevate the total lactylation level in PDLSCs. Importantly, we analyzed and demonstrated that SCO induced the lactylation of RUNX2 in PDLSCs and thus promoted osteogenic differentiation. And we also validated that the lactylation of RUNX2 at K176 site was responsible for the stabilization of RUNX2 protein through deubiquitination.

Since MSCs can be found and isolated from various tissues, stem cell therapy has got increasing attention in all kinds of regenerative therapies. As a subgroup of MSCs, PDLSCs are proven to be the cell source that is best for the regeneration of periodontal tissues.^26^ Previous studies provided evidence to show the key role of PDLSCs osteogenic differentiation in periodontal tissue regeneration.^2^, ^27^ According to the experimental data, we confirmed that SCO enhances the viability of PDLSCs, increased ALP activity and promoted mineral deposition. Therefore, our current study unveiled the promoting effect of SCO on PDLSCs osteogenic differentiation for the first time.

Lactylation is a post‐translational modification, exerting functions in various biological processes.^28^ Here, we investigated and firstly uncovered that SCO treatment led to the elevation of total lactylation levels in PDLSCs. As osteogenic differentiation is usually regulated by osteogenic genes and proteins,^29^, ^30^, ^31^, ^32^, ^33^, ^34^ we made further detection and determined that the lactylation level of RUNX2 was significantly increased in PDLSCs after SCO treatment. The changes of RUNX2 expression have been regarded to be an influence factor for the osteogenic differentiation of PDLSCs.^35^, ^36^, ^37^ We also performed rescue assays to validate the involvement of SCO‐induced RUNX2 in the osteogenic differentiation of PDLSCs. As indicated by the data of rescue assays, knockdown of RUNX2 reversed the effect of SCO treatment on the osteogenic differentiation of PDLSCs, suggesting that SCO promotes the osteogenic differentiation of PDLSCs through RUNX2.

In subsequence, we analyzed and identified the potential lactylation site K176. As shown by previous studies, the lactylation modification could enhance protein stability.^18^, ^38^ Herein, we performed the half‐life profile of RUNX2 and identified that mutation of K176 site shortened the half‐life time of RUNX2, indicating that the lactylation of RUNX2 at K176 site affected the stability of RUNX2. Ubiquitination is a post‐translational modification, which can regulate protein stability.^39^, ^40^, ^41^, ^42^ Accordingly, we conducted the ubiquitination assay and validated that the lactylation of RUNX2 at K176 site decreased the ubiquitination level of RUNX2 to stabilize RUNX2 protein.

In conclusion, our study reveals the promoting effect of the SCO‐mediated lactylation of RUNX2 on the osteogenic differentiation. Our findings indicate that SCO may contribute to the regeneration of the periodontal ligament by regulating RUNX2‐mediated PDLSCs osteogenic differentiation. However, there was still a limitation in this study. In the process of periodontal ligament regeneration, many other cells are involved, such as gingival mesenchymal stem cells. Whether scopolamine has the same effect on gingival mesenchymal stem cells, thereby promoting periodontal ligament regeneration, requires further research to confirm in the future.

## AUTHOR CONTRIBUTIONS

All authors participated in the design, interpretation of the studies and analysis of the data, and review of the manuscript. Y W drafted the work and revised it critically for important intellectual content and was responsible for the acquisition, analysis, or interpretation of data for the work; P G made substantial contributions to the conception or design of the work. All authors read and approved the final manuscript.

## FUNDING INFORMATION

This work was supported by the Guangzhou Municipal Health Science and Technology Project (grant no. 20231A011104).

## CONFLICT OF INTEREST STATEMENT

The authors declare that they have no competing interests.

## ETHICS APPROVAL AND CONSENT TO PARTICIPATE

Not applicable.

## Data Availability

The datasets used and/or analyzed during the current study are available from the corresponding author upon reasonable request.
